# Caries prevalence among schoolchildren in Zagreb, Croatia

**DOI:** 10.3325/cmj.2011.52.665

**Published:** 2011-12

**Authors:** Walter Dukić, Barbara Delija, Olga Lulić Dukić

**Affiliations:** 1 Department of Pediatric Dentistry, School of Dental Medicine, University of Zagreb, Zagreb, Croatia; 2 Dental Polyclinic Zagreb, Zagreb, Croatia

## Abstract

**Aim:**

To investigate the prevalence of dental caries and treatment needs in schoolchildren aged 7-14 years from Zagreb.

**Methods:**

Dental examinations based on the World Health Organization criteria were performed on 1168 children in the period 2009-2010. The teeth were clinically examined with standard dental instruments using visual-tactile method under standard dental light. We recorded the clinical indexes of decayed, missed, and filled teeth (DMFT and dmft; upper-case letters refer to permanent and lower-case letters to primary teeth) and decayed, missed, and filled surfaces (DMFS), as well as the significant caries index (SiC).

**Results:**

The median DMFT and DMFS of all children were 3 and 4, respectively. The median DMFT and DMFS of 12-year-old children were 4 and 5, respectively. The highest median DMFT score of 7 was found among 14-year-old children. There was a significant difference between age groups (7-10 years and 11-14 years) in DMFT and DMFS. Among 8-year-old children, the median dmft index was the highest (5.5) and SiC index was 7.4. As far as the location of caries on the surface of the first permanent molar is concerned, caries occurred mostly in the central occlusal surface (27.6%).

**Conclusion:**

Our results showed a high caries prevalence among schoolchildren in Zagreb, indicating a need for an extensive program of primary oral health care.

Dental caries is a public health problem that affects pre-school and high-school children throughout the world, leading to pain, chewing difficulties, speech problems, general health disorders, psychological problems, and lower quality of life (1-5). Although advanced preventive procedures significantly decrease the prevalence of caries in the population, fissure caries on occlusal surfaces and buccal/lingual surfaces is still a considerable problem (6). In permanent dentition, teeth with deep pits and fissures have a higher risk of caries than smooth tooth surfaces (7). Also, caries more frequently affects primary than permanent teeth due to difference in enamel structure, lack of oral hygiene, or absence of preventive measures in oral health (8). Information on caries prevalence and severity represents the basis for caries prevention programs and indicates treatment necessity in the population (9). Measures of caries prevalence are indexes of decayed (D), missed (M), and filled (F) permanent teeth (T) or surfaces (S), ie, DMFT and DMFS indexes, and decayed (d), missed (m), and filled (f) primary teeth (t) or surfaces (s), ie, dmft and dmfs indexes (10). Many reports have indicated that dental caries is decreasing in many European countries and the USA (7,11-21). With a reduction of interproximal smooth surfaces caries, an increased number of cases of occlusal caries inside pits and fissures was recorded (7). The risk of caries significantly increases among adolescents with a high frequency of cariogenic snack consumption. It is also influenced by maternal socio-economic background and educational level (11), as well as dietary, hygienic, and other socio-economic factors, which demonstrates the importance of preventive educational programs and a comprehensive caries prevention scheme for schoolchildren (12).

The aim of this study was to assess the caries prevalence in two primary schools in Zagreb using the indexes for decayed, missed, and filled teeth/surfaces for primary and permanent teeth (DMFT, DMFS, dmft) and significant caries index (SiC).

## Methods

The study was conducted in two primary schools in Zagreb, Croatia, in 2009 and 2010. The two schools were selected because of their location near the city center and proximity to each other (less than 1.5 km). According to the National Census 2001, 52% of residents of this city district had high school education and 27% had college or university education (22-24). A total of 1168 children, 583 male and 585 female, were examined for dental caries. The study was approved by the Ethics Committee of the School of Dental Medicine, in Zagreb, Croatia. The procedures and possible discomforts were explained to the children and their parents and an informed consent was obtained before the examination.

Oral examinations were performed by two specialists in pedodontics who had been validated two months before the examination. The inter-examiner reliability had a kappa value of 0.957 based on the examination of 20 children of different ages. After having assessed the completed questionnaires with general information about each child, the examination started with prophylactic cleaning of all teeth surfaces in a dental unit using standard illumination. Prophylactic cleaning was conducted using a slow rotating bristle brush on a micromotor (6000/min), with professional toothpaste Klint (Voco, Cuxhaven, Germany). Teeth were rinsed with water and air, dried, and a dry working field was made using the saliva ejector and cotton rolls to isolate teeth from buccal/labial mucosa and tongue. Examinations for dental caries were conducted using World Health Organization (WHO) criteria and procedures (25). All information was recorded on a specially printed form, including the name, age, address, school, date of examination, and dental record according to the WHO. The status of each tooth was coded using visual-tactile method for analysis. According to the WHO criteria, the dental status of primary teeth was coded with the letters from A to G (healthy tooth, filled, decayed, fissure sealing, etc). Permanent teeth were coded with the numbers from 0-9 according to the WHO codes, with 0 referring to “healthy,” 1 referring to “decayed,” etc (25). All examined teeth were included in the calculation of the final SiC, DMFT, DMFS, and dmft indexes. The indexes were compared between the age groups, male and female participants, and with the WHO criteria (26,27), which were based on the examination of 12-year-old children. The first molars were thoroughly analyzed regarding the caries localization on tooth surface (mesial, central, or distal molar surface) and WHO criteria.

The indexes were calculated as follows:

DMFS: total number of decayed (D), missing/extracted (M), or filled (F) permanent teeth surfaces (S).

DMFT: total number of decayed (D), missing (M), or filled (F) permanent teeth (T).

DMFT/DMFS: the relative relationship between DMFT and DMFS.

dmft: total number of decayed (d), missing (m) or filled (f) primary teeth (t).

SiC: the mean DMFT for the third of the population with the highest caries scores.

Chi square test was used to study the association between the prevalence of dental caries and sex and age. Kruskal-Wallis and Kolmogorov-Smirnov tests were also used. The Microsoft Office Excel 2007 for Windows (Microsoft Corporation, Redmond, WA, USA) was used for the entry of data on oral health status and for creating the charts. The data were statistically processed using SPSS 11.5 for Windows (SPSS Inc., Chicago, IL, USA).

## Results

A total of 1168 children was surveyed, 583 boys and 585 girls, with the mean age of 10.8 ± 2.3 years. Boys and girls did not significantly differ in the number of permanent teeth at a value of α = 0.05 (*P* = 0.141) and in DMFT and DMFS (*P* > 0.05).

**Figure 1:**
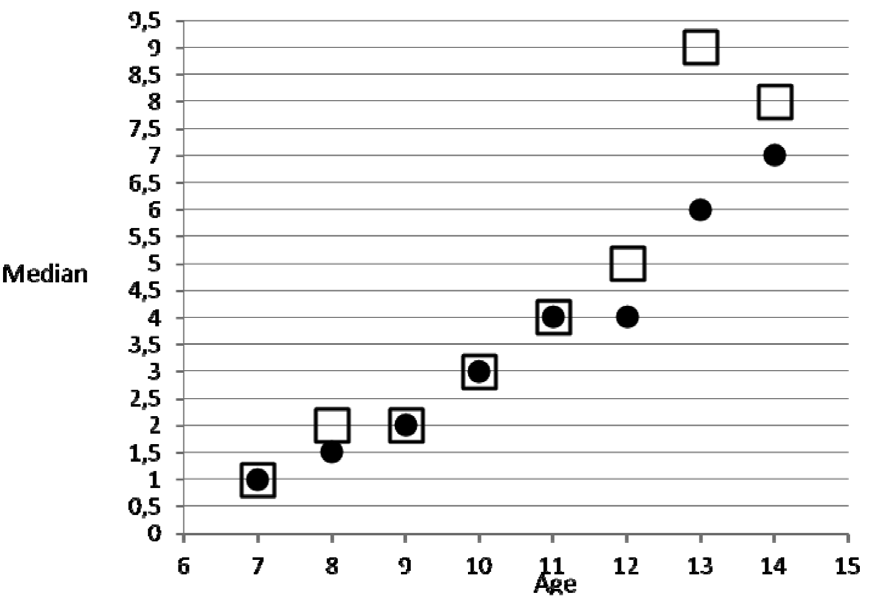
Median number of decayed, missed, and filled teeth (DMFT) (circles), decayed, missed, and filled surfaces (DMFS) (squares) according to age (N = 1168).

DMFT and DMFS increased with age (Figure 1). Children aged 14 had a mean DMFT and DMFS of 7.2 and 10.2, and median of 7 and 8, respectively (web-extra material) (web extra material 1). Children aged 12 had a mean DMFT and DMFS of 4.8 and 6.9, and median of 4 and 5, respectively. The Kruskal-Wallis test showed that older children (11-14 years) had a significantly higher DMFT (χ^2^ = 286.958 df = 1 *P* < 0.001) and DMFS (χ^2^ = 278.255 df = 1, *P* < 0.001) than younger children (7-10 years) (Table 1).

**Table 1:** Decayed, missed, and filled teeth (DMFT) and decayed, missed, and filled surfaces (DMFS) indexes according to sex and age in schoolchildren in Zagreb

		DMFT		DMFS		
	No. (%)	median	coefficient of quartile deviation	median	coefficient of quartile deviation	DMFT/DMFS × 100
**Sex:**						
male	583 (49.9)	3.00	0.71	4.00	0.78	82.9
female	585 (50.1)	3.00	0.71	4.00	0.75	84.0
**Age (years):**						
7-10	551 (47.2)	2.00*	1.00	2.00**	1.00	88.7
11-14	617 (52.8)	5.00*	0.60	6.00**	0.60	79.6
Total	1168 (100.0)	3.00	0.71	4.00	0.78	83.4

*Statistical significance (*P* < 0.001).

Kolmogorov-Smirnov test showed that both the original and transformed (logarithmic) values of DMFT and DMFS were not normally distributed. The DMFT and DMFS standard deviation had the same or even greater value than the arithmetic mean hence the use of arithmetic mean and standard deviation as descriptive indicators was not justified. Large variability in DMFT and DMFS was additionally evident from the coefficient of quartile deviation (they exceed the value of 0.6). Still, mean values ± standard deviations were calculated for comparison of caries indexes with those from similar studies. The mean DMFT for age subgroup 7-10 years was 2.0 and for the age subgroup 11-14 years it was 5.9, while the mean index of SiC for the age subgroup 7-10 years was 4.0, and for the age subgroup 11-14 years it was 10.8 (Table 2).

**Table 2:** Means and standard deviations of decayed, missed, and filled teeth (DMFT), decayed, missed, and filled surfaces (DMFS) and significant caries (SiC) indexes according to sex and age in schoolchildren in Zagreb

	N	SIC	DMFT	DMFS	DMFT/DMFS × 100
Sex:					
male	583		4.1 ± 3.9	5.8 ± 6.4	82.9
female	585		4.0 ± 4.0	5.5 ± 6.6	84.0
Age (years):					
7-10	551	4.0	2.0 ± 1.7	2.5 ± 2.6	88.7
11-14	617	10.8	5.9 ± 4.5	8.4 ± 7.6	79.6
Total	1168	7.4	4.1 ± 4.0	5.6 ± 6.5	83.4

There were 52.4% of first permanent molars with caries on occlusal surface, and only 0.84% in which the caries expanded to complete molar surface (mesial, occlusal, and distal) (Table 3). The caries occurred mostly in the central part of occlusal surface (27.6%) and it was mostly filled (26.9%) with tooth restoration (Table 4).

**Table 3:** Analysis of caries according to first permanent molar surface in schoolchildren in Zagreb

	No. (%) of children with				
Variable	without caries	partial caries (mesial, central, or distal)	complete caries (mesial + central + distal)	Total	χ^2^ test
Sex:					
male	638 (46.4)	723 (52.6)	13 (1.0)	1374	χ^2^_2_ = 0.432 *P* = 0.806
female	574 (47.1)	634 (52.1)	9 (0.8)	1217	
Age:					
7-10*	734 (51.9)	677 (47.8)	4 (0.3)	1415	χ^2^_2_ = 41.294 *P* < 0.001
11-14*	478 (40.6)	680 (57.8)	18 (1.5)	1176	
7*	73 (57.9)	53 (42.1)	-	126	χ^2^_7_ = 66.526 *P* < 0.001
8*	377 (56.3)	292 (43.6)	1 (0.1)	670	
9*	175 (49.7)	177 (50.3)	-	352	
10*	109 (40.8)	155 (58.1)	3 (1.1)	267	
11*	105 (47.3)	117 (52.7)	-	222	
12*	142 (45.8)	161 (51.9)	7 (2.2)	310	
13*	82 (35.3)	143 (61.6)	7 (3)	232	
14*	149 (36.2)	259 (62.9)	4 (0.9)	412	
Total	1212 (46.8)	1357 (52.4)	22 (0.8)	2591	

*Statistical significance (*P* < 0.001).

**Table 4:** Detailed analysis of all first molar surfaces according to World Health Organization (WHO) criteria (25)

	No. (%) of teeth with caries on		
WHO criteria	mesial molar surface	distal molar surface	central occlusal surface
Sound	4387 (93.9)	4515 (96.6)	1239 (26.5)
Decayed	134 (2.9)	57 (1.2)	1289 (27.6)
Filled, with decay	20 (0.4)	14 (0.3)	267 (5.7)
Filled, no decay	95 (2.0)	52 (1.1)	992 (21.2)
Missing, as a result of caries	17 (0.4)	17 (0.4)	17 (0.4)
Fissure sealant	0 (0)	0 (0)	852 (18.2)
Unerupted tooth (crown)	15 (0.3)	15 (0.3)	15 (0.3)
Not recorded	4 (0.08)	2 (0.04)	1 (0.02)
**Total**	**4672**	**4672**	**4672**

**Figure 2:**
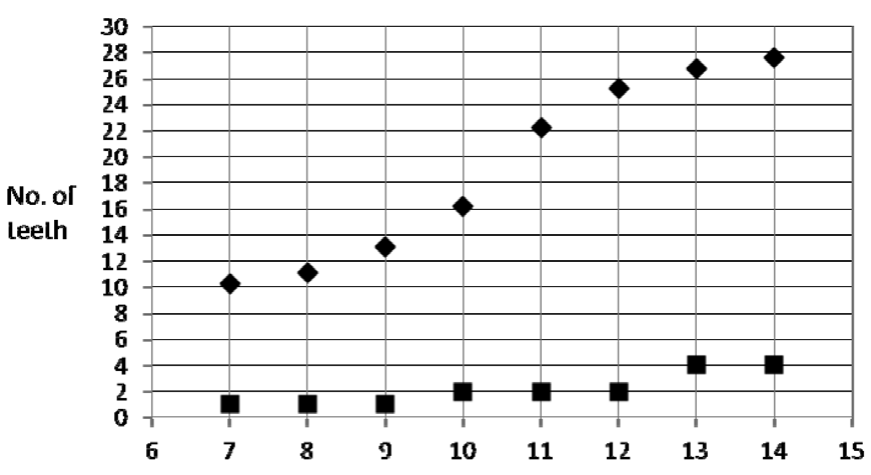
Average number of permanent teeth (rhombs) and average number of teeth with caries (squares) in children examined according to age (N = 1168).

Missing (extracted) permanent teeth (M) were very rare in this population: of 1168 children, only 51 (4.3%) were missing 1, 2, 3, 4, or 5 permanent teeth, with mean M of 0.08. Filled teeth (F) were more common, with 40.9% of children having at least one filled tooth and a mean F of 1.1. Only 24.0% of children had no caries (D), while 18.1% had one carious tooth, 15.2% had two carious teeth, 10.7% had three carious teeth, and 16.1% had four or more carious teeth. The number of carious teeth (D) increased with age (Figure 2).

There were 54.7% of all children with no caries on primary teeth, 8.7% with a single incident of caries, and 7.7% with two. There were 7.1% of children with one extracted primary tooth, 79.3% had no fillings, 9.8% only had one, 4.6% had two, and 6.3% had three or more.

## Discussion

This study showed that our population neither reached the WHO goal for 2010 of DMFT in 12-year-olds below 1, nor the one of below 3 for 2000 (26,27). The mean and median DMFT of children aged 12 years in our study was 4.8 and 4, respectively. Epidemiological indexes this high could be explained by the lack of national preventive programs, insufficient number of pediatric dentists in Croatia, and the lack of preventive and educational measures.

There are noticeable differences in the measured indexes between this and other similar studies. In surveys conducted between 1991 and 1995, the mean dmft in primary dentition in children aged 5-7 years ranged between 0.9 and 8.5 (28). It was lowest in Spain (1.0 dmft) and Denmark (1.3 dmft). National mean dmft values below 2.0 were also reported in Finland, the Netherlands, and Norway (28). Ireland had the lowest mean dmft value of 0.9 (28). All of the mentioned countries had a lower mean dmft than this study, which was 5.0 for 7-year-old children.

The mean DMFT in the majority of countries was below 3.0, and in the countries of North-Western Europe and the USA it was below 2.0 (29). However, other European populations, particularly those living in the Mediterranean area, had different results. Twelve-year-old Sicilian schoolchildren had the mean DMFT of 2.88 and their Sardinian peers of 2.4 (11,12). Twelve-year-old Greek children had a DMFT from 2.77 to 6.74, and parental education status, reason for visiting a dentist, and oral hygiene were strong determinants for caries experience (13). Spain had the mean DMFT in 12-year-old children of 1.33, with a goal to reduce it to below 1.0 by 2015 (19,20). Another study from Spain showed the mean DMFT of 2.43 in 12-year-old immigrant children and 0.99 in Spanish children (30). The mean DMFT in German 8-9-year-olds was 0.7 and in Hungary it was 0.4 (21).

On the other hand, 12-year-olds in many countries had a mean DMFT higher than 3.0, such as Latvia (7.7), Poland (5.1), Ukraine (4.4), Hungary (4.3), Lithuania and Belarus (3.8), Russia (3.7), Romania, Portugal, and Bulgaria (28). Also, Bosnia and Herzegovina, Montenegro, and Kosovo reported DMFT mean values above 3 (15-18). These results are similar to our mean DMFT of 4.8 in 12-year-old children. Considering the fact that this study was conducted in 2009-2010, this leads to a conclusion that the situation in Zagreb is not satisfactory.

The SiC index is a reliable tool for studying children with a high risk of caries (31). In Italy (Sardinia), there was a decrease in the DMFT and SiC in the period from 1989 to 2004 (32). The mean DMFT index decreased from 4.3 ± 3.1 in 1989 to 0.8 ± 1.5 in 2004 and the SiC index decreased from 7.8 (1989) to 3.9 (2004). In our study, the SiC index was extremely high, 7.4. This result indicates a great need for thoroughly planed prevention and restoration of dental caries in Croatia. However, the SiC index obtained in this study is indeed lower than that obtained in a 2008 study conducted in rural parts of Croatia on 301 children aged 3-14 years (10.89) (33). This can indicate that in the past few years a better oral hygiene and sense of oral health has been accomplished. In the same study, the mean DMFT in children aged 11-14 years was 6.7, mean DMFS 11.82, and dmft in children aged 3-6 years was 7.7. These results show a slight improvement in oral hygiene, restoration, and prevention in urban than in rural parts of Croatia. In a study that analyzed oral health in the capital cities of the former Yugoslavia in 1986, mean dmft in children aged 6 years was 8.4 and mean DMFT 0.9 (34). Mean DMFT in 6-year-old children was 0.8 and mean DMFT in 12-year-old children was 5.7 (34). The study conducted in Zagreb in 1992 reported a mean DMFT of 2.48 and 3.75 in children aged 7-8 and 9-10 years, respectively (35). Although these studies are 20-25 years old, our results showed only limited improvement in oral indexes, which is unsatisfactory for such a long time period and indicates that all new preventive and restorative measures have had limited effectiveness.

This study showed the importance of epidemiological studies for caries prevention and maintenance of oral health. The percentage of children with one or more caries lesions was too high considering the currently available preventive possibilities. The obtained DMFT and DMFS indexes, in comparison with other countries, illustrate poor oral health, low oral hygiene, and unsatisfactory prevention in Croatia, as well as a need to invest in modern preventive and therapeutic methods.
