# Supplementary material for: Caries prevalence among schoolchildren in Zagreb, Croatia
Source: Croat Med J. 2011 Dec;52(6):665–71. doi: 10.3325/cmj.2011.52.665 (PMC3243318; doi:10.3325/cmj.2011.52.665)
Supplement: Supplementary Table 1 [file CroatMedJ_52_s001.pdf]

Table 1. **Supplement.** Caries prevalence and its components in schoolchildren in Zagreb (mean±standard deviation [SD], median)

Age	Sex	N	DMFT mean±SD	DMFS mean±SD	dmft mean±SD	DMFT median	DMFS median	dmft median
7	male	20	2.3 ± 1.5	2.6 ± 2.1	5.9 ± 3.8	2.5	2.5	6.0
	female	22	1.1 ± 1.5	1.3 ± 1.8	4.1 ± 3.8	0.5	0.5	3.0
	all	42	1.8 ± 1.6	1.9 ± 2.1	5.0 ± 3.9	1.0	1.0	5.0
8	male	114	1.6 ± 1.4	1.9 ± 1.8	5.4 ± 3.5	1.0	1.0	6.0
	female	120	1.9 ± 1.7	2.3 ± 2.2	4.9 ± 3.6	2.0	2.0	5.0
	all	234	1.8 ± 1.5	2.1 ± 2.0	5.1 ± 3.6	1.5	2.0	5,5
9	male	74	2.3 ± 1.6	2.7 ± 2.4	4.3 ± 3.1	2.0	2.0	4.0
	female	76	1.9 ± 1.8	2.3 ± 2.4	3.8 ± 2.7	1.5	1.5	4.0
	all	150	2.1 ± 1.7	2.5 ± 2.4	4.0. ± 2.9	2.0	2.0	4.0
10	male	67	3.0 ± 1.8	4.5 ± 4.1	3.8 ± 2.9	3.0	3.0	4.0
	female	58	2.0 ± 1.6	2.4 ± 2.0	3.3 ± 2.8	2.0	2.0	3.0
	all	126	2.5 ± 1.8	3.5 ± 3.5	3.6 ± 2.8	3.0	3.0	4.0
11	male	59	4.0 ± 3.2	5.7 ± 5.3	1.9 ± 2.4	4.0	5.0	1.0
	female	58	3.2 ± 2.7	4.1 ± 4.3	1.2 ± 1.8	3.0	3.0	0.0
	all	117	3.6 ± 3.0	4.9 ± 4.9	1.6 ± 2.1	4.0	4.0	0.0
12	male	73	4.1 ± 3.8	6.2 ± 6.7	0.8 ± 1.8	3.0	4.0	0.0
	female	74	5.4 ± 3.8	7.6 ± 6.3	0.7 ± 1.4	4.5	6.0	0.0
	all	147	4.8 ± 3.8	6.9 ± 6.5	0.8 ± 1.6	4.0	5.0	0.0
13	male	69	7.0 ± 4.6	10.4 ± 8.2	0.2 ± 0.9	7.0	8.0	0.0
	female	67	6.7 ± 4.6	10.1 ± 7.7	0.1 ± 0.6	5.0	9.0	0.0
	all	126	6.9 ± 4.6	10.2 ± 8.0	0.2 ± 0.8	6.0	9.0	0.0
14	male	107	7.2 ± 4.8	10.1 ± 7.8	0.0 ± 0.2	6.0	8.0	0.0
	female	120	7.3 ± 4.8	10.3 ± 8.7	0.1 ± 0.3	7.0	8.5	0.0
	all	227	7.2 ± 4.8	10.2 ± 8.3	0.0 ± 0.3	7.0	8.0	0.0
Total	male	583	4.1 ± 3.9	5.8 ± 6.4	2.6 ± 3.3	3.0	4.0	0.0
	female	585	4.0 ± 4.0	5.5 ± 6.6	2.2 ± 3.1	3.0	4.0	0.0
	all	1168	4.1 ± 4.0	5.6 ± 6.5	2.4 ± 3.2	3.0	4.0	0.0

*Abbreviations: DMFT – decayed, missed, and filled permanent teeth index; DMFS – decayed, missed, and filled surfaces index; dmft – decayed, missed, and filled primary teeth index.
